# Multi-omics screening and functional validation identify SLC5A6 as a candidate disulfidptosis-related gene and prognostic biomarker in hepatocellular carcinoma

**DOI:** 10.3389/fonc.2026.1918635

**Published:** 2026-08-14

**Authors:** Chong Fu, Zejing Fang, Yanping Zhang, Wei Xu

**Affiliations:** 1 Department of Gastroenterology, Anqing Municipal Hospital, Anqing, China; 2 Graduate School, Wannan Medical University, Wuhu, China

**Keywords:** candidate disulfidptosis-related gene, disulfidptosis, hepatocellular carcinoma, SLC5A6, tumor microenvironment

## Abstract

**Objective:**

Hepatocellular carcinoma (HCC) is characterized by frequent recurrence, therapeutic resistance, and marked metabolic adaptability. Disulfidptosis is a recently described form of regulated cell death associated with glucose deprivation and disulfide stress. This study aimed to identify disulfidptosis-related genes associated with HCC progression and to investigate the potential biological role of SLC5A6.

**Methods:**

Single-cell RNA sequencing and TCGA-LIHC transcriptomic data were integrated. A literature-derived, non-directional disulfidptosis-related gene-set enrichment score was calculated using ssGSEA, and copy-number alterations were inferred using inferCNV. WGCNA, differential expression analysis, and the SLC-family gene list were integrated to identify candidate genes. Bayesian deconvolution, ESTIMATE, TIDE, and GSVA were used to evaluate tumor-microenvironment-related features and pathway signatures. The biological effects of SLC5A6 silencing were assessed using proliferation, migration, invasion, apoptosis, and xenograft assays. Glucose-deprivation-induced disulfide stress was further evaluated by measuring protein disulfide content, the NADP+/NADPH ratio, and FLNA and FLNB band patterns under non-reducing conditions.

**Results:**

Single-cell analysis showed that malignant hepatocytes with higher inferCNV-derived CNV scores exhibited greater enrichment of the disulfidptosis-related gene signature. Integration of glucose-deprivation-associated DEGs, WGCNA modules, and SLC-family genes identified SLC5A6 as a candidate disulfidptosis-related gene that was upregulated in HCC and associated with poor prognosis. Bayesian deconvolution, ESTIMATE, TIDE, and GSVA analyses linked elevated SLC5A6 expression to advanced disease, stromal and immunosuppressive cell enrichment, higher T-cell exclusion scores, and activation of Wnt/mTOR-related signaling signatures. In SLC7A11-high HCC cells, glucose deprivation increased protein disulfide content and the NADP+/NADPH ratio and altered non-reducing FLNA and FLNB band patterns, whereas these changes were partially attenuated by SLC5A6 silencing. Under conventional culture conditions, SLC5A6 silencing inhibited proliferation, migration, invasion, and xenograft growth and increased apoptosis.

**Conclusion:**

SLC5A6 is a candidate disulfidptosis-related gene and prognostic biomarker associated with malignant progression in HCC. The findings suggest that SLC5A6 may participate in glucose-deprivation-induced disulfide stress, while its direct role in regulating disulfidptotic cell death remains to be established. Its associations with immune-exclusion-related features also require further functional validation.

## 1 Introduction

Hepatocellular carcinoma (HCC) stands as a predominant cause of cancer-related mortality worldwide, imposing an escalating clinical burden. According to the most recent GLOBOCAN statistics, primary liver cancer ranks as the sixth most prevalent malignancy and the third leading cause of cancer death globally, with HCC constituting approximately 75% to 85% of all cases (1). Although surgical resection and liver transplantation are regarded as curative interventions for early-stage disease, the overall prognosis for HCC patients remains dismal, with a five-year survival rate hovering around a mere 18% (2). This elevated mortality is largely attributable to the tumor’s highly aggressive nature and substantial postoperative recurrence rates; data indicate that even following radical resection, the five-year recurrence rate remains as high as 70% (3). Furthermore, a majority of patients are diagnosed at advanced stages, thereby precluding surgical intervention. While systemic therapies such as Sorafenib and Lenvatinib have ostensibly prolonged survival, their objective response rates typically fall below 30%, and most patients develop secondary resistance within months of treatment initiation (4). Consequently, elucidating the molecular mechanisms governing HCC progression and identifying novel targets to circumvent drug resistance represent an urgent imperative in contemporary clinical research.

Evasion of regulated cell death contributes to therapeutic resistance and tumor persistence. Apoptosis and ferroptosis have been extensively investigated as cell-death mechanisms in cancer, yet tumor cells may reduce their susceptibility to these processes through alterations in survival signaling, antioxidant defenses, and metabolic adaptation (5). Such adaptations can support tumor-cell survival under therapeutic and nutrient-limiting conditions. These considerations have prompted increasing interest in additional forms of regulated cell death that may reveal previously unrecognized metabolic vulnerabilities in HCC.

Disulfidptosis is a recently described form of regulated cell death driven by intracellular disulfide stress. It is mechanistically distinct from apoptosis and ferroptosis. In SLC7A11-high cells, glucose deprivation limits NADPH availability, promotes aberrant disulfide-bond formation in actin-cytoskeletal proteins, and disrupts cytoskeletal organization, ultimately leading to cell death (6). HCC is characterized by extensive metabolic reprogramming and increased reliance on glycolysis (7), suggesting that disulfidptosis-related disulfide stress may be relevant to the survival and adaptation of HCC cells under nutrient-limiting conditions. However, the biological significance of this process in HCC and the molecular factors that modulate the associated stress response remain incompletely understood.

The solute carrier (SLC) superfamily occupies a pivotal position within the regulatory network of disulfidptosis. As the largest family of transporter proteins in the human genome, comprising over 400 members, SLCs mediate the transmembrane transport of glucose, amino acids, and inorganic ions (8). Altered expression of SLC-family genes contributes to cancer metabolic reprogramming and may influence tumor-cell adaptation to nutrient-limited environments. Given the close relationship between disulfidptosis, SLC7A11-mediated cystine uptake, and glucose-dependent NADPH homeostasis, specific SLC members may modulate disulfidptosis-related stress responses in HCC. However, the SLC genes associated with these responses and their functional relevance to HCC progression remain incompletely defined. In this study, we established a glucose-deprivation-induced disulfide stress model in SLC7A11-high HCC cells and performed transcriptomic sequencing to characterize the associated molecular changes. By integrating model-derived differentially expressed genes with WGCNA modules, a literature-derived disulfidptosis-related gene set, and the SLC-family gene list, we identified SLC5A6 as a candidate disulfidptosis-related gene associated with poor prognosis in HCC. Single-cell RNA sequencing and Bayesian deconvolution were subsequently used to examine the associations between SLC5A6-high malignant-cell state and tumor-microenvironment-related features. Finally, *in vitro* and *in vivo* functional assays were performed to evaluate the tumor-promoting role of SLC5A6, while additional biochemical analyses assessed its potential involvement in glucose-deprivation-induced disulfide stress. Together, these findings provide a basis for further investigation of SLC5A6 in HCC metabolic stress responses and malignant progression.

## 2 Materials and methods

### 2.1 Data acquisition and preprocessing

This study utilized the GSE166635 single-cell dataset and the TCGA-LIHC cohort. Single-cell RNA sequencing data and bulk transcriptomic data for HCC were retrieved from the Gene Expression Omnibus (GEO) (9) and The Cancer Genome Atlas (TCGA) repositories (10), respectively. For the scRNA-seq data, quality control was rigorously performed using the Seurat package in R (11, 12). Low-quality cells, characterized by excessive mitochondrial gene content or aberrant gene detection counts, were systematically excluded. The remaining high-quality cells underwent normalization via the NormalizeData function, followed by the identification of highly variable genes using FindVariableFeatures. Subsequently, dimensionality reduction and clustering analyses were executed utilizing RunPCA and RunUMAP. Cell type annotation was predicated on the expression profiles of canonical marker genes. Furthermore, a comprehensive list of SLC family members was curated from the study by Lewis et al (13) (**Supplementary Table 1**).

### 2.2 Disulfidptosis-related signature score and copy number variation (CNV) analysis

A literature-derived set of ten disulfidptosis-related genes, including GYS1, NDUFS1, OXSM, LRPPRC, NDUFA11, NUBPL, NCKAP1, RPN1, SLC3A2, and SLC7A11, was curated from a previous experimental study (6). The complete gene list, biological annotations, inclusion criteria, and supporting evidence are provided in **Supplementary Table 2**. These genes were treated as a non-directional disulfidptosis-related signature, and single-sample gene set enrichment analysis (ssGSEA) was performed using the GSVA package (14), with the parameters set as follows: method = “ssgsea”, min.sz = 1, and max.sz = 500. The resulting score represents the relative enrichment of the disulfidptosis-related transcriptional signature rather than a direct measure of disulfidptosis activity or cellular sensitivity. Genomic instability was assessed by inferring CNV in malignant cells using the inferCNV R package (15). Immune cells served as the reference baseline to calculate CNV scores across distinct cell subpopulations.

### 2.3 Weighted gene co-expression network analysis (WGCNA)

A weighted gene co-expression network was constructed utilizing the WGCNA R package (16). Initially, sample correlations were calculated to identify and exclude outliers. Subsequently, an optimal soft-thresholding power (β) was determined to establish a scale-free network topology. Genes were clustered into distinct co-expression modules based on the Topological Overlap Measure (TOM). Key modules were identified by evaluating the correlation between the Module Eigengene (ME) and the disulfidptosis score. Genes exhibiting both high Module Membership (MM) and Gene Significance (GS) were selected as hub genes.

### 2.4 Inference of cell subpopulation abundance via bayesian deconvolution

To categorize malignant cells based on *SLC5A6* expression, we extracted the log-normalized expression values of *SLC5A6* across the entire malignant cell population. We calculated the median of these expression values to serve as the stratification threshold. Cells exhibiting expression levels surpassing this median were defined as the *SLC5A6-high malignant-cell state(Malignant_SLC5A6_high)*, whereas those with expression levels at or below the threshold were designated as the *SLC5A6-low malignant-cell state(Malignant_SLC5A6_low)*. Subsequently, to quantify the abundance of these specific malignant subpopulations within the bulk TCGA-LIHC cohort, we employed a Bayesian deconvolution algorithm (17). A single-cell reference expression matrix was constructed using the expression profiles of the SLC5A6-high and SLC5A6-low malignant-cell states, together with those of the major immune and stromal cell populations. Based on this reference matrix, TCGA-LIHC bulk transcriptomic data were deconvoluted to estimate the relative abundance of the SLC5A6-high malignant-cell state in each patient sample. The main parameters were set as key = NULL, outlier.cut = 0.01, prism.cau = 2, outlier.fraction = 0.1, and n.cores = 5. Final cell-type fractions were extracted using which.theta = “final” and state.or.type = “type”. The resulting cell-abundance matrix is provided in **Supplementary Table 4**.

### 2.5 Tumor immune microenvironment assessment and immunotherapy response prediction

To evaluate the immune and stromal status within the tumor microenvironment (TME), we adopted the ESTIMATE algorithm (18). Utilizing bulk RNA-seq expression profiles from the TCGA-LIHC cohort, we calculated the Immune Score, Stromal Score, and ESTIMATE Score for each sample using the estimate package in R. Furthermore, to predict potential patient responses to immune checkpoint blockade (ICB) therapy, we leveraged the TIDE (Tumor Immune Dysfunction and Exclusion) computational framework (http://tide.dfci.harvard.edu/) (19). We uploaded the normalized gene expression matrix to the TIDE online platform to compute the TIDE Score, T-cell Dysfunction Score, and T-cell Exclusion Score. Concurrently, the algorithm inferred the relative abundance of immunosuppressive cell types, including M2-polarized tumor-associated macrophages (TAM-M2) and myeloid-derived suppressor cells (MDSCs).

### 2.6 Establishment of a glucose-deprivation-induced disulfide stress model

Human hepatocellular carcinoma cell lines (Bel-7404, Huh7, HepG2, SMMC-7721, QGY-7701, and Hep3B) were procured from the Cell Bank of the Chinese Academy of Sciences (Shanghai, China).All cell lines were routinely maintained in DMEM (Gibco, USA, Cat. No. C11965500BT) supplemented with 10% fetal bovine serum (Gibco, USA, Cat. No. A3160802) and 1% penicillin-streptomycin solution (Gibco, USA, Cat. No. 15140122) within a humidified incubator at 37 °C with 5% CO_2_. Upon reaching 80%-90% confluence, cells were washed with PBS (Gibco, USA, Cat. No. C10010500BT) and dissociated using 0.25% trypsin (BIOMEDICAL, China, Cat. No. B2048) for subculturing. Basal SLC7A11 expression across the six HCC cell lines was descriptively evaluated at both the mRNA and protein levels using RT-qPCR and Western blotting, respectively. QGY-7701 and SMMC-7721 cells were selected for subsequent glucose-deprivation experiments because they consistently exhibited relatively high basal SLC7A11 expression in both assays. Pairwise statistical significance among the individual cell lines was not used as a selection criterion. To establish the *in vitro* glucose-deprivation-induced disulfide stress model and assess cell viability, cells in the logarithmic growth phase were harvested via trypsinization and resuspended in complete medium. The cell suspension was adjusted to a density of 5*10^4^ cells/mL and seeded into 96-well plates (100 μL per well) with triplicate wells for each group. The plates were pre-incubated for 24 hours to ensure complete adherence. Subsequently, the spent medium was aspirated, and the cells were washed twice with PBS. The experimental group was transitioned to glucose-free DMEM, while the control group received glucose-containing complete medium. Following a four-hour glucose starvation period, 10 μL of Cell Counting Kit-8 (CCK-8) solution (REMARKABLE, China, Cat. No. B1099) was added to each well, ensuring no bubbles were introduced. After a further two-hour incubation, absorbance at OD value was measured using a microplate reader to evaluate cell viability.

### 2.7 Determination of protein disulfide content

Cells were harvested and lysed by adding the corresponding extraction buffer (Solarbio, China, Cat. No. BC5795), followed by ultrasonic disruption in an ice bath. The lysate was centrifuged at 3000 rpm for 10 minutes at 4 °C, after which the supernatant was discarded and the pellet retained. The precipitate was dissolved in Reagent I and diluted five-fold. Sample aliquots were mixed with Powder I and allowed to react for 30 minutes with the cap open, followed by the sequential addition of the extraction buffer and Reagent II, and subsequent centrifugation to collect the supernatant. The supernatant was mixed with Reagent III to measure the initial absorbance (A1); subsequently, Reagent IV was added, and after a 10-minute incubation, the final absorbance (A2) was recorded at a wavelength of 412 nm. The disulfide content was calculated based on the difference in absorbance and normalized against protein concentration determined via the BCA assay.

### 2.8 Determination of NADP+/NADPH ratio

Intracellular levels of NADP+ and NADPH were quantified utilizing the NADP+/NADPH Assay Kit (Beyotime, China, Cat. No. S0179). Cell lysates were harvested and subjected to centrifugation at 12,000 × g for 10 minutes at 4 °C. The resultant supernatant was aliquoted into two portions: one was designated for the determination of total NADP+/NADPH, while the other was incubated at 60 °C for 30 minutes to specifically isolate and detect NADPH. Samples were incubated with G6PDH working solution at 37 °C in the dark for 10 minutes, followed by the addition of WST-8 colorimetric solution for a subsequent incubation period of 10–20 minutes. Absorbance was measured at 450 nm to calculate the NADP+/NADPH ratio, which was subsequently normalized against protein concentrations determined via the BCA assay.

### 2.9 Transcriptome sequencing (RNA-seq) and differential gene expression analysis

To investigate transcriptomic alterations associated with glucose-deprivation-induced disulfide stress, QGY-7701 and SMMC-7721 cell lines were selected and stratified into a Control group (Ctrl, maintained in normal culture medium) and a Model group (Model, subjected to glucose-free culture conditions). Two independent biological replicates were prepared for each cell line under each condition, resulting in a total of 8 RNA-sequencing samples: two cell lines × two treatment conditions × two biological replicates. Upon harvest, cell samples were preserved on dry ice and transported to Beijing Biomarker Technologies Co., Ltd. for transcriptome sequencing. Briefly, total RNA was extracted utilizing TRIzol reagent, with quality control assessments performed via NanoDrop 2000 and the Agilent 2100 Bioanalyzer. Qualified RNA samples were utilized to construct cDNA libraries, which were subsequently sequenced on the Illumina platform using the PE150 strategy. Raw data underwent rigorous quality control and filtration to yield Clean Data (Q30 > 94.89%), which was then aligned to the human reference genome (GRCh38) using HISAT2. Gene-level raw read counts were obtained for each sample and used as input for differential expression analysis in edgeR. Differentially expressed genes were identified using the thresholds of |log2FC| ≥ 1 and FDR < 0.05. The raw sequencing data and processed datasets generated in this study have been deposited in the NCBI Gene Expression Omnibus (GEO) under the accession number GSE330217.

### 2.10 Cell culture and transfection

To silence *SLC5A6* expression, specific small interfering RNA (si-SLC5A6) or a negative control (si-NC) was transfected into hepatocellular carcinoma cells utilizing NanoTrans 40™ (BIOMEDICAL, China, RK001002). Cells were seeded into 6-well plates; upon achieving 60%–80% confluence, the culture medium was replaced with fresh complete medium 30 minutes prior to transfection. A transfection complex was generated by diluting 100 pmol of siRNA and 3 μL of NanoTrans separately in 250 μL of serum-free DMEM, mixing the solutions, and incubating the mixture at room temperature for 15 minutes before dropwise addition to the cell monolayers. The medium was replaced with complete medium 8 hours post-transfection. Cells were harvested 48 hours later, and knockdown efficiency was corroborated via RT-qPCR and Western blot analysis.

### 2.11 Cell migration and invasion assays

Migratory potential was assessed utilizing the wound healing assay. Cell monolayers in 6-well plates, upon reaching >90% confluence, were vertically scored using a 10 μL pipette tip. Non-adherent cells were removed via triple lavage with PBS (Gibco, USA, C10010500BT), and the medium was replaced with serum-free medium. Photomicrographs of wound closure were acquired at 0, 12, and 24 hours using fluorescence microscopy to calculate migration rates. Invasive capacity was evaluated via Transwell assays. The upper chambers of the Transwell inserts were pre-coated with Matrigel (BD, USA, 356234) diluted 1:8 and allowed to polymerize overnight at 37 °C. A suspension of 6×10^4^ cells in 100 μL of serum-free medium was seeded into the upper chamber, while 600 μL of medium supplemented with 20% FBS (Gibco, USA, A3160802) was added to the lower chamber to serve as a chemoattractant. Following a 48-hour incubation, non-invading cells on the upper surface were removed; cells that had invaded the lower surface were fixed with 4% paraformaldehyde for 30 minutes, stained with 0.1% crystal violet for 30 minutes, and quantified in randomly selected fields under a microscope.

### 2.12 Cell apoptosis assay

Apoptosis rates were quantified utilizing the Annexin V-FITC/PI Apoptosis Detection Kit (Beyotime, China, C1062S). Following treatment, harvested cells were washed with PBS and resuspended in 195 μL of binding buffer. Sequential addition of 5 μL Annexin V-FITC and 10 μL Propidium Iodide (PI) was performed; the suspension was gently agitated and incubated in the dark at ambient temperature (20-25 °C) for 20 minutes. The reaction was immediately terminated by placing the samples in an ice bath, followed by flow cytometric analysis.

### 2.13 Lentiviral infection and stable cell line construction

To establish stable cell lines exhibiting either knockdown or overexpression of *SLC5A6*, a lentiviral transduction system was employed. Hepatocellular carcinoma cells in the logarithmic growth phase were seeded into 6-well plates and infected upon reaching 15%–30% confluence. In accordance with the optimal multiplicity of infection (MOI) determined via preliminary experiments, the lentiviral suspension was mixed with Polybrene (Beyotime, China, C0351) and introduced to the culture wells. The medium was replaced with fresh complete medium 12 hours post-infection. Antibiotic selection was initiated 48–72 hours after infection and maintained until complete mortality of the control group was observed, yielding stable transfectants for subsequent *in vivo* and *in vitro* experimentation.

### 2.14 Non-reducing western blot analysis

QGY-7701 and SMMC-7721 cells were divided into six groups: Ctrl, Model, siSLC5A6, Model + siSLC5A6, Model + TCEP, and siSLC5A6 + TCEP. The Ctrl group was cultured in complete medium containing glucose; the Model group was cultured in glucose-free medium; the siSLC5A6 group consisted of SLC5A6-silenced cells cultured in complete medium; the Model + siSLC5A6 group consisted of SLC5A6-silenced cells subjected to glucose deprivation; the Model + TCEP group was subjected to glucose deprivation and treated with TCEP; and the Model + siSLC5A6 + TCEP group consisted of SLC5A6-silenced cells subjected to glucose deprivation and TCEP treatment. Cells in the Model groups were cultured in glucose-free DMEM for 4 h, whereas the corresponding control groups were maintained in glucose-containing medium. For the combined-treatment groups, cells were transfected with siSLC5A6 before glucose deprivation, and TCEP was administered at a final concentration of 1 mM.QGY-7701 and SMMC-7721 cells were lysed in RIPA buffer (Solarbio, China, Cat. No. R0010) supplemented with 1 mM PMSF. After centrifugation, protein concentrations were determined using a BCA assay (Beyotime, China, Cat. No. P0010). Equal amounts of protein were mixed with non-reducing loading buffer without dithiothreitol or β-mercaptoethanol, separated by 10% SDS–PAGE, and transferred onto PVDF membranes (Millipore, USA, Cat. No. IPVH00010). The membranes were incubated with antibodies against FLNA, FLNB, and β-actin, followed by HRP-conjugated secondary antibodies. Protein bands were detected using enhanced chemiluminescence, and the approximately 280-kDa FLNA and FLNB bands were quantified and normalized to β-actin. All experiments were repeated three times.

### 2.15 Xenograft tumor model

All animal procedures received ethical approval from the Ethics Committee of Anhui Medical University (Anhui,China,No.LLSC20252926). A total of 10 Male BALB/c nude mice (4–6 weeks old), procured from Shanghai SLAC Laboratory Animal Co., Ltd., were maintained under specific pathogen-free (SPF) conditions. The mice were randomly allocated into two groups (n = 5 per group).Stable transfectants (SLC5A6 knockdown groups and controls) in the logarithmic growth phase were harvested via trypsinization and resuspended in PBS to a final density of 5* 10^7^ cells/mL. A volume of 200 μL of the cell suspension was inoculated subcutaneously into the nude mice using a sterile syringe. Twenty-six days post-inoculation, mice were euthanized via CO2 inhalation in an induction chamber. In accordance with American Veterinary Medical Association (AVMA) guidelines, the CO2 flow rate displaced 30% to 70% of the chamber volume per minute. Death was confirmed by cessation of respiration and heartbeat, followed by cervical dislocation as a secondary physical method to ensure death. During the outcome assessment, including tumor excision and weighing, investigators were blinded to the group allocation. No animals were excluded from the final analysis. Representative tissue specimens were fixed in 4% paraformaldehyde for subsequent histopathological examination.

### 2.16 Hematoxylin and eosin (H&E) staining

Fixed tumor tissues underwent gradient ethanol dehydration, xylene clearing, and paraffin embedding before being sectioned at 4 μm. Following deparaffinization in xylene and gradient rehydration in ethanol, sections were stained using an H&E Staining Kit (Servicebio, China, G1003). The protocol involved hematoxylin staining for 3–5 minutes, differentiation and bluing in tap water, followed by eosin staining for 5 minutes. Sections were subsequently dehydrated, cleared, and mounted with neutral balsam for morphological observation via optical microscopy, where nuclei appeared blue and cytoplasm appeared red.

### 2.17 Immunohistochemistry (IHC)

To assess tumor cell proliferation, Ki-67 expression was evaluated via immunohistochemistry. Paraffin sections were baked at 66 °C, deparaffinized, rehydrated, and subjected to high-pressure antigen retrieval in citrate buffer (pH 6.0; ebiogo, China, B009). Upon cooling, sections were incubated with 3% H_2_O_2_ to block endogenous peroxidase activity. The sections were incubated with the primary Ki-67 antibody (Bioss, China, No. bsm-60738R) at 37 °C for 60 minutes, followed by incubation with the secondary antibody (ebiogo, China, B001) at 37 °C for 30 minutes. Visualization was achieved using a DAB chromogen solution (ebiogo, China, B011), with hematoxylin used for nuclear counterstaining. Images were acquired under a microscope, with brownish-yellow staining indicating Ki-67 positive nuclei. Nuclei showing brownish-yellow staining were scored as Ki-67 positive, and the Ki-67 labeling index (%) was calculated as the number of Ki-67-positive nuclei divided by the total number of nuclei × 100%.

### 2.18 Statistical analysis

All statistical analyses were performed using GraphPad Prism software. Quantitative data are expressed as the mean ± standard deviation (SD). Comparisons between the two groups were performed using an unpaired Student’s t-test for normally distributed data, or the Mann-Whitney U test for data not adhering to a normal distribution. A P-value of less than 0.05 was considered statistically significant.

## 3 Results

### 3.1 Construction of the single-cell atlas and characterization of disulfidptosis features in hepatocellular carcinoma

**Figure 1:**
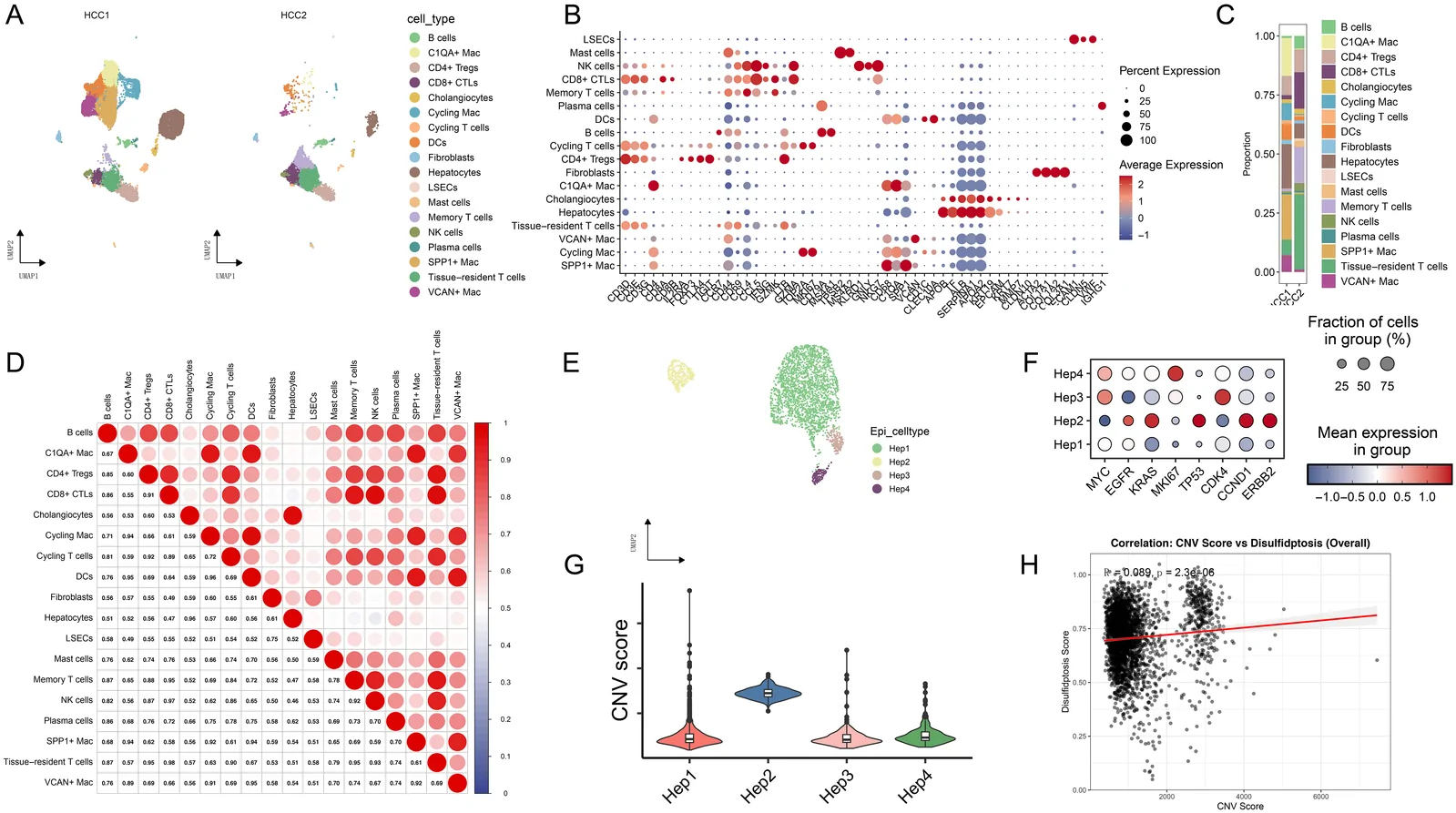
**(A)** A UMAP dimensionality reduction plot elucidating the clustering of predominant cell phenotypes within the HCC tumor microenvironment. **(B)** A bubble plot delineating the expression landscapes of canonical marker genes across major cellular subpopulations. **(C)** A stacked bar chart exemplifying the proportional stratification of cell lineages across distinct sample cohorts. **(D)** An inter-cellular correlation heatmap revealing the reciprocal relationships in gene expression patterns among microenvironmental subsets. **(E)** UMAP re-clustering analysis of the hepatocyte compartment, distinguishing four subpopulations with divergent transcriptional architectures. **(F)** A bubble plot displaying the expression intensity and prevalence of critical oncogenes and cell cycle indices within specific hepatocyte subsets. **(G)** A violin plot illustrating the distribution of CNV scores among hepatocyte clusters. **(H)** A scatter plot analysis evidencing a positive correlation between CNV scores and disulfidptosis scores.

To gain profound insights into the heterogeneity of the TME in HCC and its potential nexus with disulfidptosis, we initiated our study with a comprehensive analysis of single-cell transcriptomic data. UMAP dimensionality reduction visualized the predominant cell lineages within the TME, comprising T cells, B cells, macrophages, fibroblasts, and hepatocytes (**Figure 1A**). The fidelity of these cell subpopulation annotations was rigorously validated via a bubble plot depicting the expression of canonical marker genes (**Figure 1B**). Subsequent analysis of cellular proportions unveiled marked heterogeneity in cellular composition across distinct sample cohorts (**Figure 1C**), highlighting substantial inter-patient variability in immune cell landscape patterns.

To further corroborate the precision of subpopulation annotations and elucidate transcriptomic relationships among distinct cell types, we performed Pearson correlation analysis (**Figure 1D**). The resulting heatmap demonstrated that cell subpopulations sharing a common lineage exhibited high transcriptomic similarity and clustered cohesively, whereas distinct lineages displayed significantly lower correlations. This distinct modular clustering pattern attests to the high biological specificity and reliability of our cell segmentation and annotation methodologies.

To interrogate the characteristics of malignant cells, we performed re-clustering of the hepatocyte population, identifying four subpopulations with distinct transcriptional profiles (**Figure 1E**). Expression profiling of pivotal oncogenes and cell cycle markers revealed that the Hep2 subpopulation exhibited the most pronounced malignant phenotype (**Figure 1F**). Crucially, CNV analysis demonstrated that the Hep2 subpopulation possessed the highest CNV score, further corroborating its hyper-malignant nature (**Figure 1G**). Moreover, scatter plot analysis indicated a significant positive correlation between CNV scores and disulfidptosis-related gene-set enrichment score (**Figure 1H**). This association may reflect transcriptional changes related to chromosomal instability, metabolic stress adaptation, cell-cycle activity, or tumor subclonal heterogeneity. However, as both scores were computationally derived from transcriptomic data, the observed correlation should not be interpreted as evidence of causality.

### 3.2 Establishment of a glucose-deprivation-induced disulfide stress model in hcc cells and transcriptomic characterization

**Figure 2:**
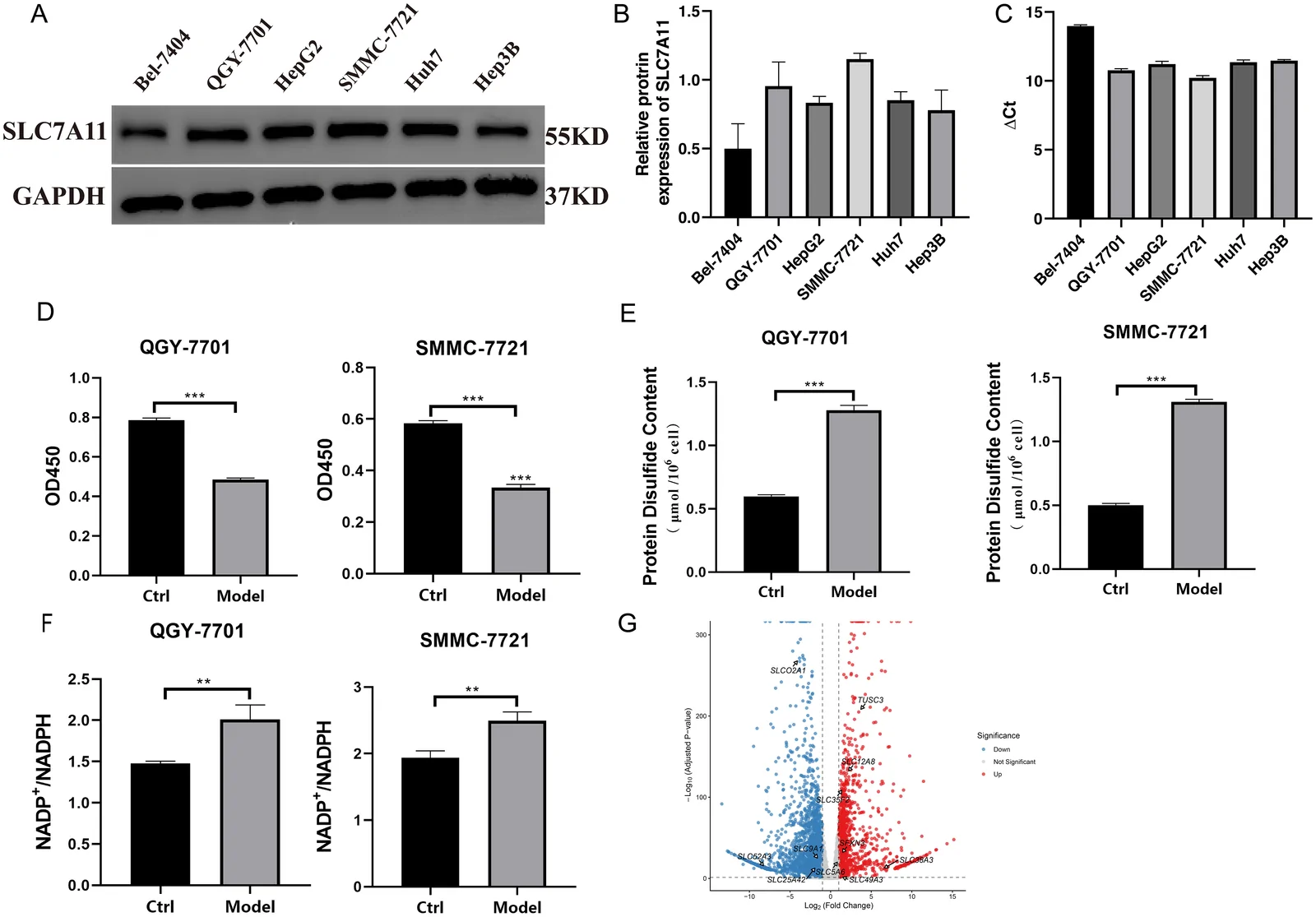
**(A, B)** Western blot analysis accompanied by densitometric quantification delineating the basal expression profiles of the SLC7A11 protein across six distinct HCC cell lines (Bel-7404, Huh7, HepG2, SMMC-7721, QGY-7701, Hep3B). **(C)** RT-qPCR assays determining the relative transcriptional abundance of SLC7A11 mRNA within the respective HCC cell lineages. **(D)** Cell viability assessments utilizing the CCK-8 assay to contrast the Control group (Ctrl) against the glucose-deprived cohort (Model). **(E)** Comparative quantification of intracellular protein disulfide bond accumulation in the control versus model groups. **(F)** Evaluation of the intracellular NADP+/NADPH redox ratio distinguishing the model group from the control. **(G)** A volcano plot visualization highlighting DEGs arising from the comparison between the model and control groups. (***p < 0.001, **p < 0.01).

To establish a glucose-deprivation-induced disulfide stress model for transcriptomic analysis, we first assessed the basal expression of SLC7A11 across six HCC cell lines using Western blotting and RT-qPCR. QGY-7701 and SMMC-7721 cells exhibited relatively high SLC7A11 protein and mRNA expression levels (**Figures 2A–C**) and were therefore selected for model establishment. Following glucose deprivation, CCK-8 assays showed a significant reduction in cell viability compared with cells maintained in glucose-containing medium (**Figure 2D**). Glucose deprivation also markedly increased intracellular protein disulfide content and the NADP+/NADPH ratio in both cell lines (**Figures 2E, F**), indicating the induction of disulfide stress and redox imbalance. Transcriptomic sequencing was subsequently performed to characterize the molecular changes associated with this model. Differential expression analysis identified 4,380 DEGs, including 1,663 upregulated and 2,717 downregulated genes in the glucose-deprived group (**Figure 2G**). The complete normalized gene-expression dataset is provided in **Supplementary Table 3**. Given that only two biological replicates were included for each cell line under each condition, the statistical power of the RNA-seq analysis was limited, particularly for detecting modest expression changes. Accordingly, the identified DEGs were treated as exploratory candidates for subsequent integrative screening rather than as independently validated glucose-deprivation-responsive genes.

### 3.3 WGCNA-based identification of key SLC family genes associated with disulfidptosis

**Figure 3:**
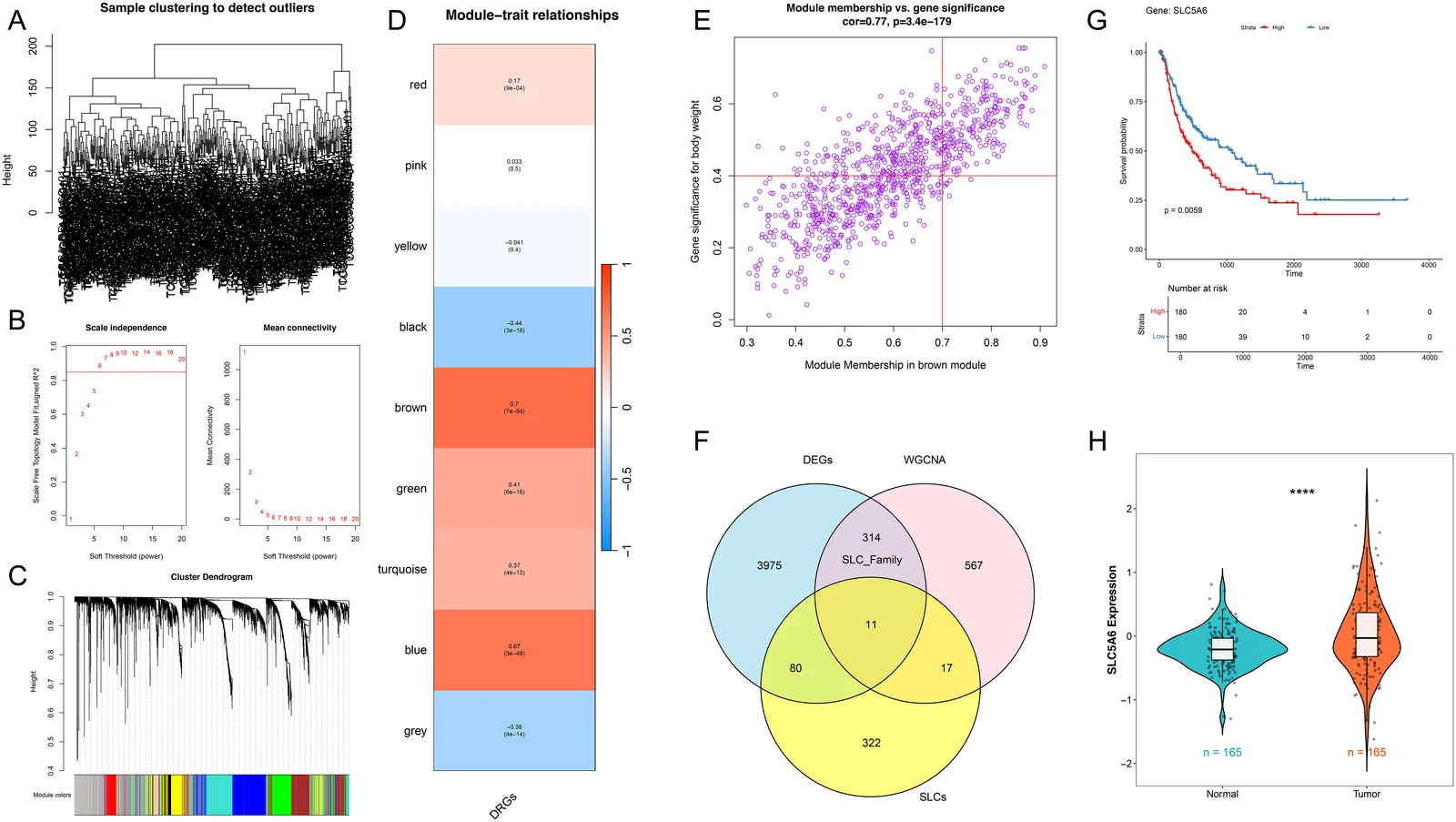
**(A)** A sample clustering dendrogram utilized for the detection of outlier specimens. **(B)** Network topology analysis displaying the scale-free fit index (left) and mean connectivity (right) as a function of soft-thresholding power. **(C)** A gene clustering dendrogram constructed via the TOM, delineating identified co-expression modules. **(D)** A heatmap visualizing module-trait relationships, where inscribed values represent correlation coefficients and associated P-values. **(E)** A scatter plot correlating MM with GS within pivotal modules. **(F)** A Venn diagram illustrating the intersection between DEGs, key WGCNA module genes, and the SLC family gene set. **(G)** Kaplan-Meier curves estimating OS stratified by high versus low SLC5A6 expression cohorts. **(H)** Violin plots contrasting SLC5A6 expression levels between tumor and normal tissues. (****p < 0.0001).

To identify pivotal SLC family genes regulating disulfidptosis, we constructed a WGCNA network based on the TCGA dataset. With a soft-thresholding power of β=6 (R^2^>0.85) and specific clustering parameters (minModuleSize=50, deepSplit=2, cut height=0.25), a total of 9 co-expression modules were identified (**Figures 3A–D**). Module-trait association analysis revealed that the Brown module exhibited the most significant positive correlation with disulfidptosis features (DRGs) (r=0.77, p<0.001), suggesting that this module aggregates key regulatory genes governing disulfidptosis (**Figure 3E**). By intersecting the Brown module, the DEGs, and the SLC family gene list, we screened 11 overlapping candidate genes (**Figure 3F**), all of which were labeled in the volcano plot in **Figure 2G**. Subsequent survival analysis and expression validation pinpointed SLC5A6 as the core target: its high expression was significantly associated with poor patient prognosis (**Figure 3G**) and was markedly elevated in tumor tissues (**Figure 3H**).

### 3.4 The inferred abundance of the SLC5A6-high malignant-cell state is associated with immune-exclusion-related features

**Figure 4:**
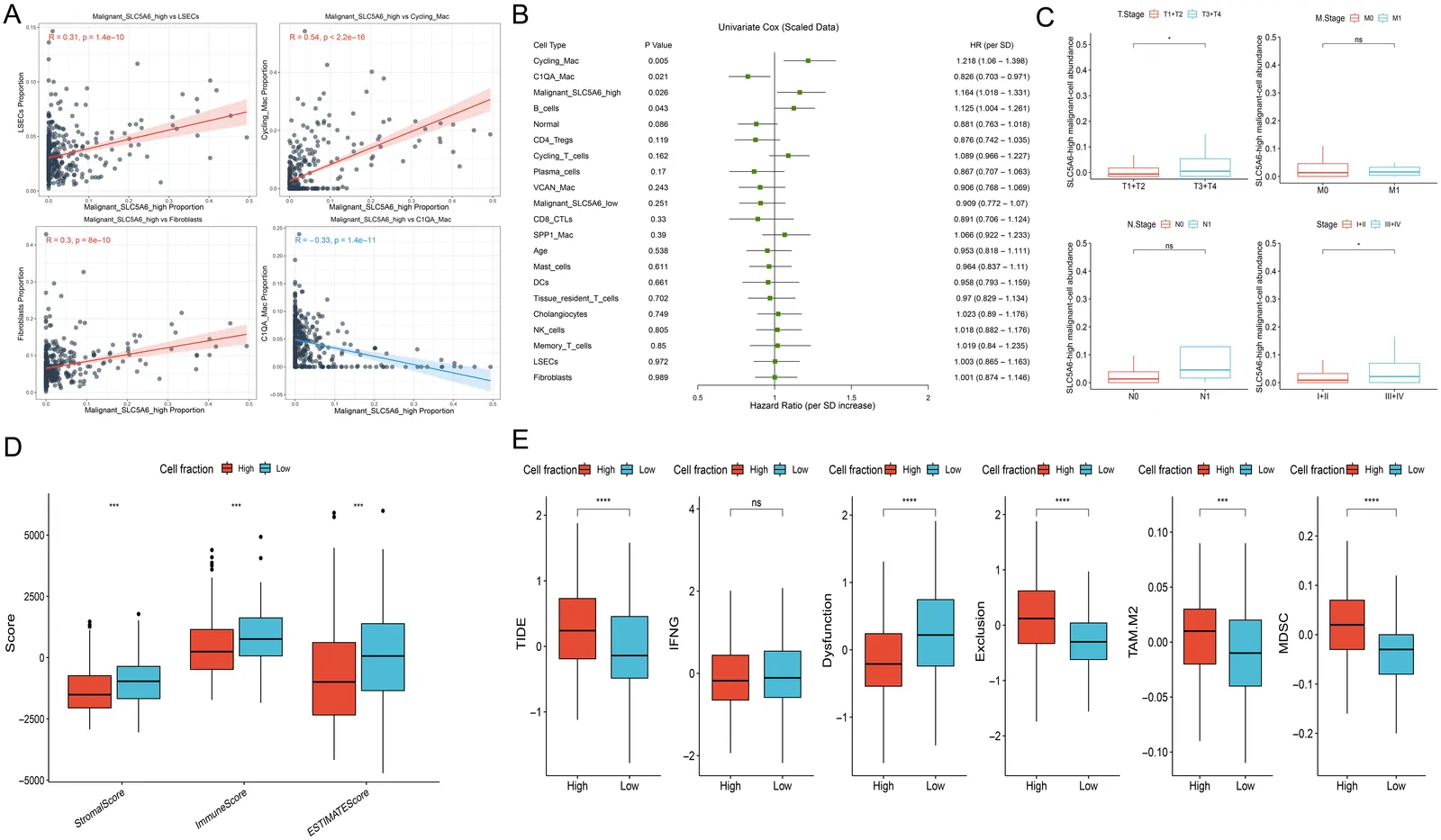
**(A)** scatter plots showing the correlations between SLC5A6-high malignant-cell abundance and key immune and stromal components, including C1QA+ macrophages and fibroblasts. **(B)** A forest plot summarizing the univariate Cox regression analysis for distinct cell subpopulations within the TCGA-LIHC cohort. **(C)** Box plots showing the inferred abundance of the SLC5A6-high malignant-cell state across clinicopathological subgroups defined by T stage, M stage, N stage, and overall pathological stage.**(D)** Comparative evaluation of immune microenvironment scores derived via the ESTIMATE algorithm.**(E)** Comparisons of TIDE-derived immune-evasion metrics, including the TIDE score, IFNG signature, T-cell dysfunction score, T-cell exclusion score, TAM.M2 score, and MDSC score, between the high- and low-abundance groups. (ns, not significant; *P < 0.05, **P < 0.01, ***P < 0.001, and ****P < 0.0001).

To examine the associations between the SLC5A6-high malignant-cell state and the tumor microenvironment, we inferred the abundance of cellular subpopulations within the TCGA-LIHC cohort utilizing a Bayesian deconvolution algorithm (**Supplementary Table 4**) and conducted correlation and prognostic analyses. Correlation analysis showed that the inferred abundance of the SLC5A6-high malignant-cell state was positively associated with liver sinusoidal endothelial cells and fibroblasts and showed a negative correlation trend with C1QA+ macrophages (**Figure 4A**). Univariate Cox regression analysis further identified the inferred abundance of the SLC5A6-high malignant-cell state (HR = 1.164, P = 0.026) and Cycling Macrophages (HR = 1.218, P = 0.005) as significant prognostic risk factors, whereas C1QA+ macrophages emerged as protective factors (HR = 0.826, P = 0.021) (**Figure 4B**). Moreover, a higher inferred abundance of the SLC5A6-high malignant-cell state was significantly associated with advanced T stage and overall pathological stage, indicating that this expression-defined malignant-cell state was more abundant in patients with advanced disease (**Figure 4C**).

To further characterize the molecular and immune features associated with this state, TCGA-LIHC samples were divided into high- and low-abundance groups according to the inferred abundance of the SLC5A6-high malignant-cell state. ESTIMATE analysis showed that the high-abundance group had significantly higher Stromal and Immune Scores (**Figure 4D**). TIDE analysis showed that the high-abundance group had a significantly higher T-cell Exclusion Score, whereas no corresponding increase in the Dysfunction Score was observed (**Figure 4E**). Together with the higher Stromal and Immune Scores, this result suggested an immune-infiltrated but exclusion-related tumor state. Cellular-composition analysis further indicated higher inferred abundances of TAM-M2 cells and MDSCs in the high-abundance group. GSVA further revealed enrichment of pathway signatures related to DNA replication, mismatch repair, homologous recombination, and the cell cycle, together with Wnt, mTOR, Notch, and ErbB signaling signatures, in the high-abundance group (**Supplementary Figure 1**). Because Wnt/β-catenin and mTOR signaling have previously been associated with immune-exclusion-related processes (20, 21), their enrichment may provide a possible biological context for the immune characteristics observed in this group.

### 3.5 Knockdown of SLC5A6 inhibits proliferation, migration, and invasion while inducing apoptosis in HCC cells

**Figure 5:**
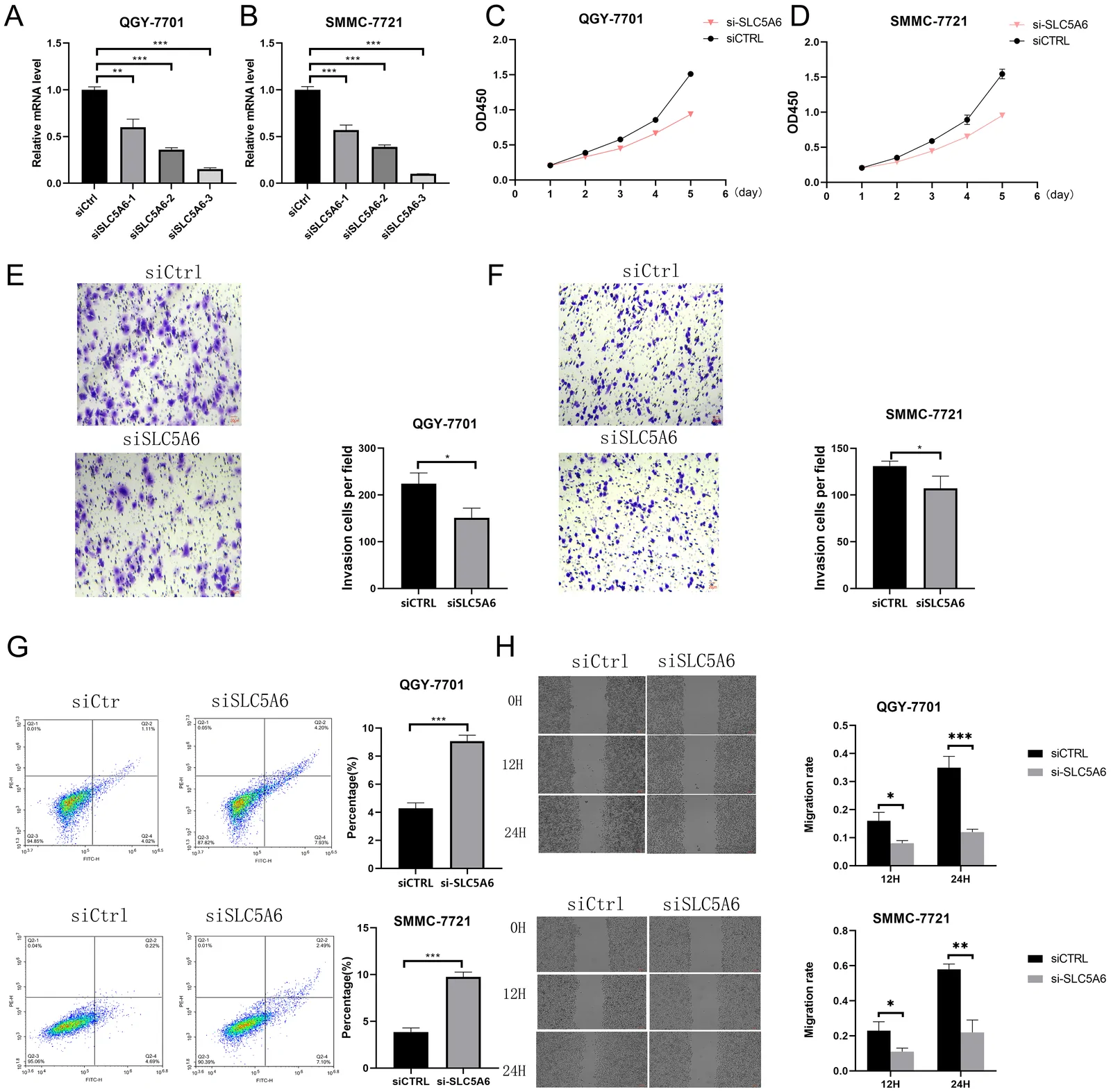
**(A, B)** RT-qPCR analysis validating the knockdown efficiency of SLC5A6 in QGY-7701 and SMMC-7721 cell lines. **(C, D)** CCK-8 proliferation assays contrasting the growth kinetics of the control (siCtrl) and knockdown (siSLC5A6) groups within QGY-7701 and SMMC-7721 cells. **(E, F)** Representative photomicrographs and quantitative analyses of transwell invasion assays, illustrating the impact of SLC5A6 silencing on the invasive potential of QGY-7701 and SMMC-7721 cells. **(G)** Flow cytometric evaluation of apoptosis rates following SLC5A6 knockdown in QGY-7701 and SMMC-7721 cells; the adjacent bar chart depicts the quantitative statistical analysis of apoptotic ratios. **(H)** Wound healing assays (0 h, 12 h, and 24 h) and corresponding quantitative assessments evaluating cellular migratory capabilities. (*p < 0.05, **p < 0.01, ***p < 0.001).

To delineate the biological function of *SLC5A6*, we transfected specific siRNAs into QGY-7701 and SMMC-7721 cells, with RT-qPCR results corroborating the efficacy of *SLC5A6* knockdown (**Figures 5A, B**). Functional assays demonstrated that silencing *SLC5A6* markedly suppressed the proliferative capacity of HCC cells (**Figures 5C, D**). Transwell assays revealed a pronounced attenuation in the invasive potential of the knockdown group (**Figures 5E, F**). Flow cytometric analysis indicated a significant augmentation in the apoptotic rate following *SLC5A6* knockdown (**Figure 5G**). Furthermore, wound healing assays evidenced that the *SLC5A6* knockdown group exhibited substantially reduced wound closure rates at 12 and 24 hours compared to the control group, indicative of compromised migratory capability (**Figure 5H**). Collectively, these *in vitro* data underscore the critical role of *SLC5A6* in sustaining the malignant phenotype of HCC cells.

### 3.6 Knockdown of SLC5A6 suppresses HCC tumor growth *in vivo*

**Figure 6:**
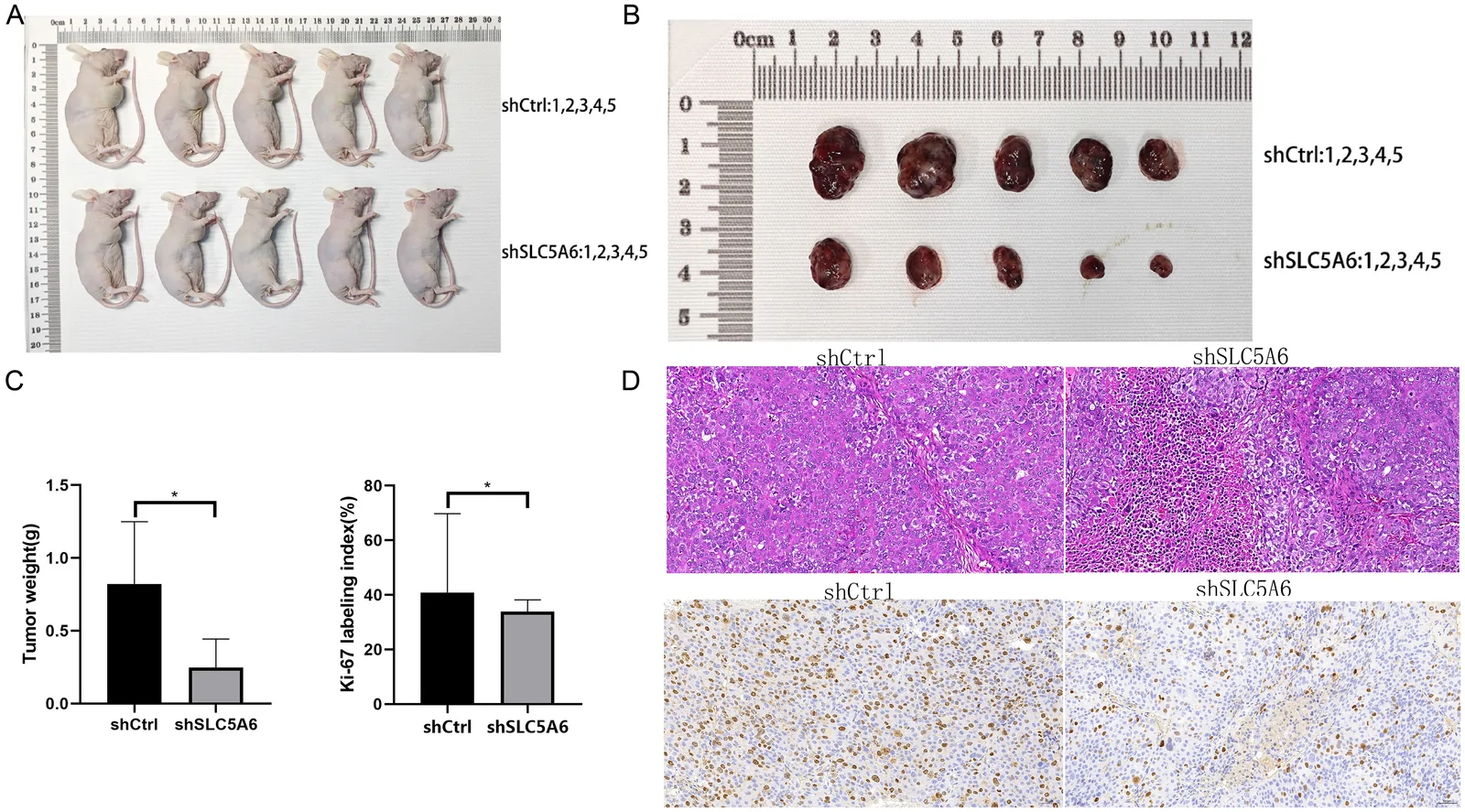
**(A, B)** Representative imagery illustrating the nude mouse xenograft tumorigenesis models and the gross macroscopic morphology of resected tumor masses. **(C)** Quantification of tumor weights and the Ki-67 positive rate (%) in each group at the experimental endpoint. **(D)** Representative images of H&E staining and Ki-67 immunohistochemistry in tumor sections. (*p < 0.05).

Finally, we substantiated the *in vivo* functionality of *SLC5A6* utilizing a nude mouse subcutaneous xenograft model. Representative *in vivo* imaging and macroscopic photographs of excised tumors depicted that tumor volumes in the *SLC5A6* knockdown group (shSLC5A6) were significantly smaller than those in the control group (shCtrl) (**Figures 6A, B**). Quantitative analysis confirmed a marked reduction in tumor mass in the knockdown cohort (**Figure 6C**). Histological examination (H&E staining and Ki-67 immunohistochemistry) further revealed significantly diminished cell proliferative activity within the tumor tissues of the knockdown group (**Figures 6C, D**). In conclusion, both *in vitro* and *in vivo* experiments consistently attest to the pivotal oncogenic role of *SLC5A6* in the genesis and progression of HCC.

### 3.7 SLC5A6 silencing attenuates glucose-deprivation-induced disulfide stress

**Figure 7:**
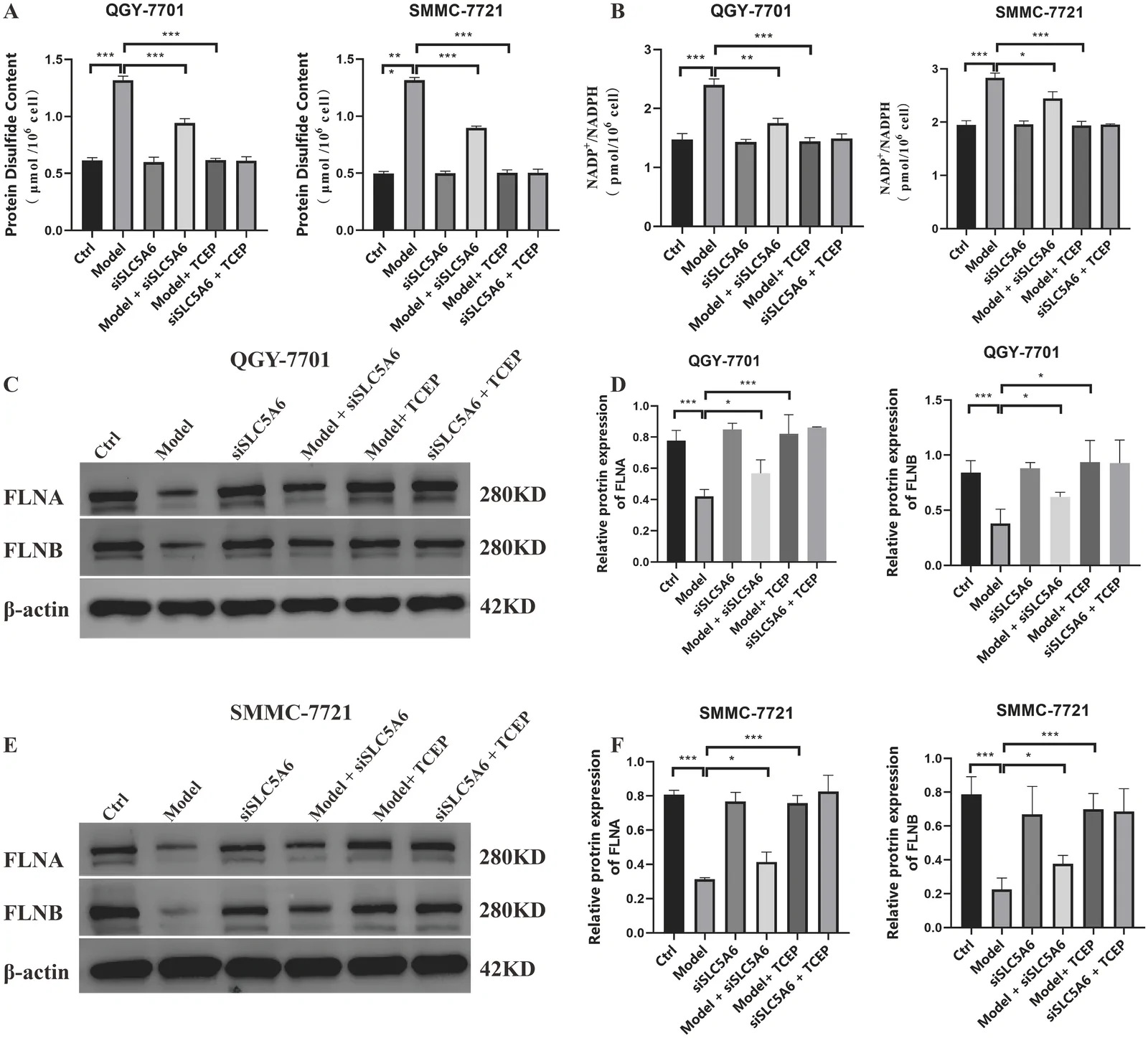
**(A)** Intracellular protein disulfide content in QGY-7701 and SMMC-7721 cells under the indicated treatment conditions. **(B)** NADP+/NADPH ratios in QGY-7701 and SMMC-7721 cells. **(C, D)** Representative non-reducing western blot images of FLNA and FLNB and the corresponding densitometric quantification in QGY-7701 cells. **(E, F)** representative non-reducing western blot images of FLNA and FLNB and the corresponding densitometric quantification in SMMC-7721 cells. β-actin was used as the loading control.(*p < 0.05, **p < 0.01, ***p < 0.001).

To further examine the potential involvement of SLC5A6 in glucose-deprivation-induced disulfide stress, intracellular protein disulfide content and the NADP+/NADPH ratio were evaluated in QGY-7701 and SMMC-7721 cells. Glucose deprivation significantly increased total protein disulfide content and the NADP+/NADPH ratio in both cell lines. SLC5A6 knockdown partially attenuated these changes, while TCEP treatment also reduced the glucose-deprivation-induced increases in these indicators (**Figures 7A, B**). Non-reducing Western blotting was subsequently performed to examine disulfide stress-associated alterations in the actin-cytoskeletal proteins FLNA and FLNB. Under non-reducing conditions, glucose deprivation markedly reduced the FLNA and FLNB band intensities in both QGY-7701 and SMMC-7721 cells. SLC5A6 silencing partially restored the monomeric FLNA and FLNB signals under glucose-deprivation conditions. A similar recovery of the detectable monomeric bands was observed following TCEP treatment (**Figures 7C–F**).

## 4 Discussion

The clinical management of HCC remains challenging because of its metabolic plasticity and aggressive behavior. Through integrated multi-omics screening, we identified SLC5A6 as a candidate disulfidptosis-related gene associated with poor prognosis in HCC. Under conventional culture conditions, SLC5A6 silencing reduced cell proliferation, migration, and invasion and increased apoptosis, while stable SLC5A6 knockdown suppressed xenograft tumor growth. To further explore the link between SLC5A6 and disulfidptosis-related stress, we examined glucose-deprivation-induced redox and cytoskeletal protein alterations in the SLC7A11-high HCC cell lines QGY-7701 and SMMC-7721. Under glucose-replete conditions, SLC5A6 silencing did not significantly affect the NADP+/NADPH ratio, total protein disulfide content, or the monomeric FLNA and FLNB band intensities detected under non-reducing conditions. By contrast, glucose deprivation increased the NADP+/NADPH ratio and protein disulfide content and reduced the detectable monomeric FLNA and FLNB signals. These changes were partially attenuated by either SLC5A6 silencing or TCEP treatment, whereas combined SLC5A6 silencing and TCEP treatment under glucose-replete conditions produced no additional detectable effects. Together, these observations suggest that the influence of SLC5A6 on disulfide homeostasis becomes more apparent under glucose-limiting conditions. Because redox imbalance, protein disulfide accumulation, and alterations in actin-cytoskeletal proteins are characteristic features associated with disulfidptosis, our findings support SLC5A6 as a candidate disulfidptosis-related gene involved in glucose-deprivation-induced disulfide stress. Whether SLC5A6 directly regulates the complete disulfidptosis program or more broadly modulates cellular adaptation to metabolic and redox stress remains to be established.

Our bioinformatic analyses revealed associations between the inferred abundance of the SLC5A6-high malignant-cell state and immune-exclusion-related features of the tumor microenvironment. TCGA-LIHC samples with a higher inferred abundance of this malignant-cell state exhibited higher ESTIMATE Immune Scores. However, this increase was not accompanied by an apparent survival benefit and was observed together with more advanced clinicopathological characteristics. Integration of TIDE analysis and cell-composition estimation further showed that the high-abundance group had higher T-cell exclusion scores and higher inferred abundances of immunosuppressive cell populations. These findings suggest that a greater inferred abundance of the SLC5A6-high malignant-cell state is associated with a stromal-rich and immunosuppressive tumor state consistent with immune exclusion, rather than a simple immune-desert phenotype (22).

GSVA further showed that the high-abundance group exhibited greater enrichment of Wnt, mTOR, Notch, and ErbB pathway signatures. Aberrant Wnt/β-catenin signaling has been reported to limit dendritic-cell recruitment and effector T-cell infiltration (23), whereas mTOR signaling may influence metabolic competition within the tumor microenvironment and impair T-cell function (24, 25). These previously reported mechanisms provide a plausible biological context for the co-occurrence of these pathway signatures and the elevated T-cell exclusion score. However, the present analyses do not demonstrate that SLC5A6 directly activates these pathways or that Wnt/mTOR signaling mediates the observed immune-associated features.

At the cellular level, correlation analysis showed that the inferred abundance of the SLC5A6-high malignant-cell state was associated with several stromal and immunosuppressive components of the tumor microenvironment. A higher inferred abundance of this state was positively associated with fibroblast and liver sinusoidal endothelial cell abundance and coincided with a higher Stromal Score in the high-abundance group. These stromal features may provide a biological context for the observed immune-exclusion-related phenotype (26). Concurrently, cellular-composition analysis indicated higher inferred abundances of TAM-M2 cells and MDSCs, together with a lower inferred abundance of C1QA+ macrophages, in the high-abundance group. These cellular features were consistent with a relatively immunosuppressive tumor microenvironment. Although TAM-M2 cells and MDSCs have been reported to suppress antitumor immune responses, the present analysis does not demonstrate that the SLC5A6-high malignant-cell state directly regulates the abundance or function of these populations (27, 28).

Taken together, these findings support a model in which elevated SLC5A6 expression is linked to a metabolically active, stromal-rich, and immunosuppressive tumor state. The concomitant enrichment of Wnt- and mTOR-related signaling signatures, fibroblasts, and immunosuppressive cell populations may favor the development of an immune-exclusion-related microenvironment, thereby contributing to the association between SLC5A6 expression and unfavorable clinical outcomes in HCC. Future functional studies will be needed to define the precise role of SLC5A6 in this regulatory network.

Several limitations should be acknowledged. Although SLC5A6 silencing attenuated glucose-deprivation-induced protein disulfide accumulation, the increase in the NADP+/NADPH ratio, and alterations in the non-reducing FLNA and FLNB band patterns, these findings primarily reflect changes in disulfide stress, which represents a central biochemical component of disulfidptosis. Thus, our results support SLC5A6 as a candidate disulfidptosis-related gene involved in glucose-deprivation-induced disulfide stress, but its direct role in regulating disulfidptotic cell death remains to be established. Further studies assessing cell-death rescue, SLC7A11 dependence, alternative cell-death pathways, and actin-cytoskeletal disulfide crosslinking will be required to determine whether SLC5A6 alters cellular susceptibility to disulfidptosis. In addition, the associations between SLC5A6 expression, Wnt/mTOR-related signaling, and immune microenvironment features were inferred primarily from transcriptomic and computational analyses and require validation at the protein and functional levels. Moreover, the xenograft experiments were performed in nude mice and therefore could not evaluate T-cell-dependent immune exclusion or immunotherapy response. Future studies incorporating pathway perturbation, co-culture systems, spatial profiling, and immunocompetent animal models will help clarify the roles of SLC5A6 in disulfidptosis-related stress responses and tumor–immune interactions.

## 5 Conclusion

This study identifies SLC5A6 as a candidate disulfidptosis-related gene and prognostic biomarker associated with malignant progression in HCC. Functional experiments showed that SLC5A6 silencing suppressed cell proliferation, migration, invasion, and xenograft tumor growth, supporting a tumor-promoting role for SLC5A6. Under glucose-deprivation conditions, SLC5A6 silencing attenuated the increase in the NADP+/NADPH ratio and protein disulfide content and partially restored the non-reducing FLNA and FLNB signals. These findings link SLC5A6 to disulfidptosis-associated disulfide stress, although its direct role in regulating disulfidptotic cell death remains to be established. Multi-omics analyses further revealed associations between elevated SLC5A6 expression, Wnt/mTOR-related signaling signatures, and stromal and immunosuppressive features of the tumor microenvironment. Further mechanistic studies are required to determine whether SLC5A6 directly influences disulfidptosis and tumor–immune interactions.

## Data Availability

The datasets presented in this study can be found in online repositories. The names of the repository/repositories and accession number(s) can be found in the article/**Supplementary Material**.

## Supplementary material

Supplementary Figure 1 Enlarged high-resolution heatmap of KEGG pathway activity associated with the abundance of the SLC5A6-high malignant-cell state in HCC. TCGA-LIHC samples were stratified into high- and low-abundance groups according to the median inferred abundance of the SLC5A6-high malignant-cell state. Rows represent KEGG pathways, whereas columns represent individual tumor samples. The annotation bars at the top indicate the inferred abundance of the SLC5A6-high malignant-cell state and the corresponding group assignment. GSVA enrichment scores were standardized by row and displayed as z-scores ranging from −2 to 2. Pathways were hierarchically clustered according to their enrichment patterns. *P < 0.05, **P < 0.01, and ***P < 0.001 for comparisons between the high- and low-abundance groups.
