# Supplementary material for: Multi-omics screening and functional validation identify SLC5A6 as a candidate disulfidptosis-related gene and prognostic biomarker in hepatocellular carcinoma
Source: Front Oncol. 2026 Aug 14;16:1918635. doi: 10.3389/fonc.2026.1918635 (PMC13521847; doi:10.3389/fonc.2026.1918635)
Supplement: Supplementary file 1 [file DataSheet1.docx]

**Supplementary Figure 1 | Enlarged high-resolution heatmap of KEGG pathway activity associated with the abundance of the SLC5A6-high malignant-cell state in HCC.**TCGA-LIHC samples were stratified into high- and low-abundance groups according to the median inferred abundance of the SLC5A6-high malignant-cell state. Rows represent KEGG pathways, whereas columns represent individual tumor samples. The annotation bars at the top indicate the inferred abundance of the SLC5A6-high malignant-cell state and the corresponding group assignment. GSVA enrichment scores were standardized by row and displayed as z-scores ranging from −2 to 2. Pathways were hierarchically clustered according to their enrichment patterns. *P < 0.05, **P < 0.01, and ***P < 0.001 for comparisons between the high- and low-abundance groups.
